# Machine learning based on clinico-biological features integrated ^18^F-FDG PET/CT radiomics for distinguishing squamous cell carcinoma from adenocarcinoma of lung

**DOI:** 10.1007/s00259-020-05065-6

**Published:** 2020-10-15

**Authors:** Caiyue Ren, Jianping Zhang, Ming Qi, Jiangang Zhang, Yingjian Zhang, Shaoli Song, Yun Sun, Jingyi Cheng

**Affiliations:** 1 Department of Nuclear Medicine, Shanghai Proton and Heavy Ion Center, Fudan University Cancer Hospital, Shanghai, 201321 China; 2 Department of Nuclear Medicine, Shanghai Proton and Heavy Ion Center, Shanghai, 201315 China; 3 Shanghai Engineering Research Center of Proton and Heavy Ion Radiation Therapy, Shanghai, China; 4 Department of Oncology, Shanghai Medical College, Fudan University, Shanghai, 200032 China; 5 Center for Biomedical Imaging, Fudan University, Shanghai, 200032 China; 6 Shanghai Engineering Research Center for Molecular Imaging Probes, Shanghai, 200032 China; 7 Department of Research and Development, Shanghai Proton and Heavy Ion Center, Shanghai, 201321 China

**Keywords:** Squamous cell carcinoma, Adenocarcinoma, ^18^F-FDG PET/CT, Radiomics, Nomogram, Machine learning

## Abstract

**Purpose:**

To develop and validate a clinico-biological features and ^18^F-fluorodeoxyglucose (FDG) positron emission tomography/computed tomography (PET/CT) radiomic-based nomogram via machine learning for the pretherapy prediction of discriminating between adenocarcinoma (ADC) and squamous cell carcinoma (SCC) in non-small cell lung cancer (NSCLC).

**Methods:**

A total of 315 NSCLC patients confirmed by postoperative pathology between January 2017 and June 2019 were retrospectively analyzed and randomly divided into the training (*n* = 220) and validation (*n* = 95) sets. Preoperative clinical factors, serum tumor markers, and PET, and CT radiomic features were analyzed. Prediction models were developed using the least absolute shrinkage and selection operator (LASSO) regression analysis. The performance of the models was evaluated and compared by the area under receiver-operator characteristic (ROC) curve (AUC) and DeLong test. The clinical utility of the models was determined via decision curve analysis (DCA). Then, a nomogram was developed based on the model with the best predictive efficiency and clinical utility and was validated using the calibration plots.

**Results:**

In total, 122 SCC and 193 ADC patients were enrolled in this study. Four independent prediction models were separately developed to differentiate SCC from ADC using clinical factors-tumor markers, PET radiomics, CT radiomics, and their combination. The DeLong test and DCA showed that the Combined Model, consisting of 2 clinical factors, 2 tumor markers, 7 PET radiomics, and 3 CT radiomic parameters, held the highest predictive efficiency and clinical utility in predicting the NSCLC subtypes compared with the use of these parameters alone in both the training and validation sets (AUCs (95% CIs) = 0.932 (0.900–0.964), 0.901 (0.840–0.957), respectively) (*p* < 0.05). A quantitative nomogram was subsequently constructed using the independently risk factors from the Combined Model. The calibration curves indicated a good consistency between the actual observations and nomogram predictions.

**Conclusion:**

This study presents an integrated clinico-biologico-radiological nomogram that can be accurately and noninvasively used for the individualized differentiation SCC from ADC in NSCLC, thereby assisting in clinical decision making for precision treatment.

**Electronic supplementary material:**

The online version of this article (10.1007/s00259-020-05065-6) contains supplementary material, which is available to authorized users.

## Introduction

Non-small cell lung cancer (NSCLC) accounts for approximately 85% of lung cancer that is the most common cause of cancer-related mortality worldwide, with an estimated 1.4 million deaths each year [1]. Adenocarcinoma (ADC) and squamous cell carcinoma (SCC) are the most common subtypes of NSCLC [2]. Different pathological subtypes have distinct phenotypic and biological characteristics, which are directly related to the clinical treatment and outcome [3–5]. With advances in targeted therapies, molecularly targeted agents that inhibit epidermal growth factor receptor (EGFR) and anaplastic lymphoma kinase (ALK) can significantly improve the efficacy and reduce the toxicity of NSCLC, as almost all these gene mutations are found in ADC [6, 7]. Therefore, accurately predicting the histological subtypes is essential for determining better therapeutic strategies in NSCLC.

An invasive biopsy for histological confirmation is commonly used in clinical practice [8]. However, with the development of various detection technologies in recent years, high-precision noninvasive detection has been paid more attention and recognized by clinicians; moreover, biopsy is contraindicated for patients with severe cardiopulmonary insufficiency, such as severe pulmonary arterial hypertension, or uncorrectable coagulopathy, or unable to cooperate with the operation [9, 10]. In addition, when the pathological tissue obtained from the first puncture is few and fails to meet the needs for an accurate diagnosis, it is more difficult to biopsy again [11]. Thus, it is clinically important and necessary to explore a reliable, noninvasive, and practical method for the pre-therapy prediction of the histologic subtypes for treatment decision making and prognosis estimation in NSCLC patients.

Radiomics based on conventional medical images has been used to quantitatively assess tumor heterogeneity in more detail than visual analysis by analyzing the distribution and relationship of pixel or voxel gray levels in the lesion area [12, 13]. ^18^F-fluorodeoxyglucose (FDG) positron emission tomography/computed tomography (PET/CT)–based radiomics have been shown to have potential in differentiating ADC from SCC [14, 15]. Further studies have revealed that the discrimination performance could be further improved by combining with clinical features, like sex and smoking history (area under curve (AUC) = 0.859), which are higher than that of radiomic alone [16, 17]. However, only PET radiomic parameters were extracted and analyzed in the above studies. There is no single feature that can adequately describe the pathological phenotype of lesions due to the tumor heterogeneity [18].

Hence, the aim of this study was to develop and validate a prediction model, integrating the clinical characteristics, tumor marker levels [19], and radiomic features extracted from both the PET and CT images from the same volume of interest (VOI), for differentiating SCC from ADC in NSCLC and to provide a visually quantitative nomogram in clinical practice.

## Materials and methods

### Patients

**Fig. 1:**
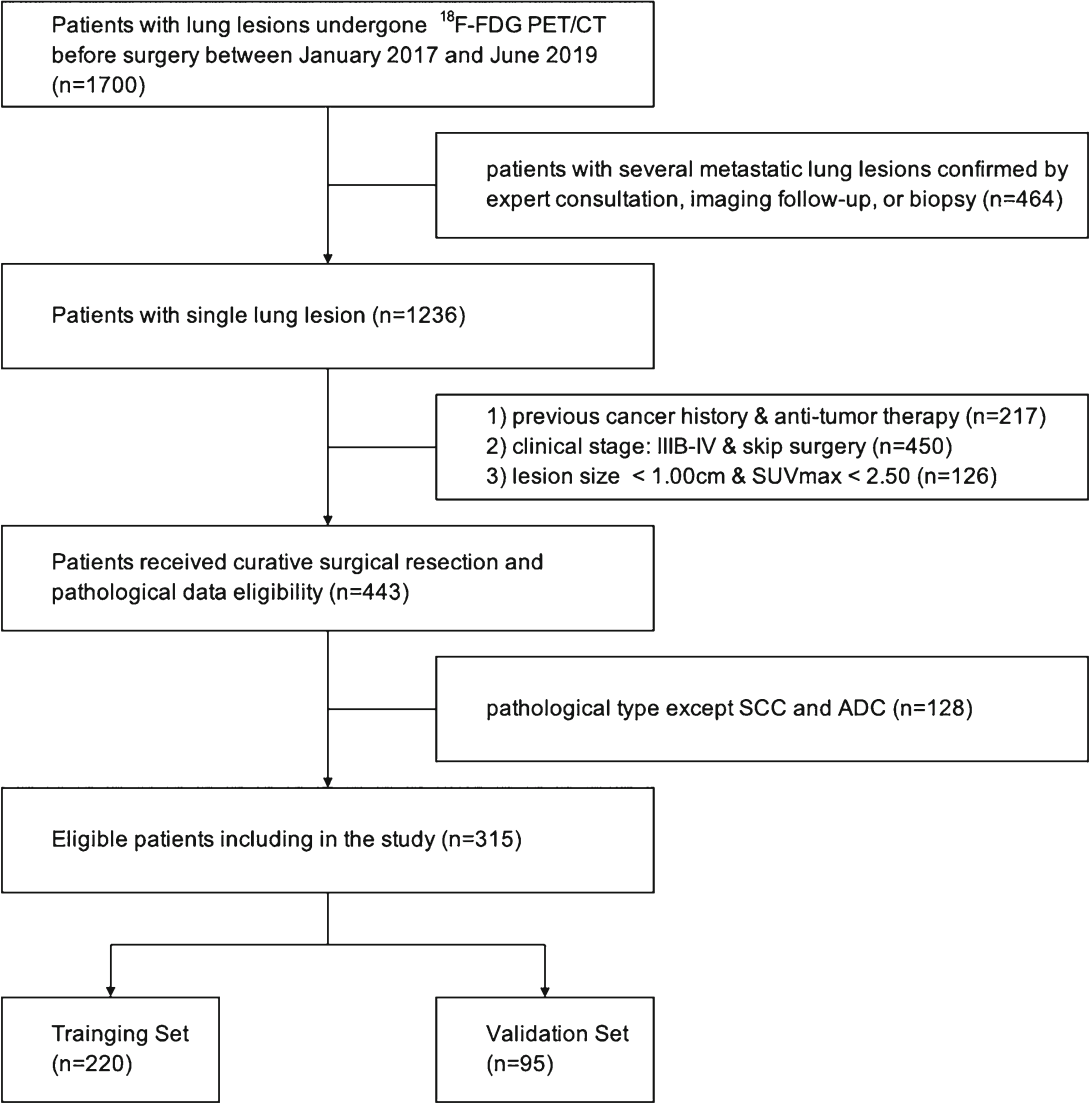
Flow chart showing the patient selection and exclusion

We conducted a retrospective analysis of records from patients with NSCLC who were diagnosed by curative surgical resection between January 2017 and June 2019. This retrospective study was approved by the ethics committee of Shanghai Proton and Heavy Ion Center, and the requirement for informed consent was waived. The inclusion criteria included the following: (1) ADC or SCC that was confirmed by postoperative pathology according to the 2015 World Health Organization (WHO) classification [20], (2) standard routine whole-body PET/CT less than 30 days before surgery, and (3) single lesion with maximum standardized uptake value (SUVmax) > 2.50 and size > 1.00 cm. The exclusion criteria included the following: (1) previous history of malignant tumors and (2) anti-tumor therapy before PET/CT examination. We excluded 1385 patients among the 1700 patients with lung lesions initially recruited in our cancer center’s database to ensure the relationship between single pathological subtype and baselined clinico-biologico-radiological features. The patient recruitment process is presented in Fig. 1.

Finally, totally 315 consecutive NSCLC patients were enrolled in this study, comprising 200 males and 115 females (mean age, 61.89 ± 9.10 years, range, 27–84 years), as summarized in Table 1. Patients were randomly divided into two independent sets according to a 7:3 ratio: training set (*n* = 220) and validation set (*n* = 95). Baseline data pertaining to clinical characteristics, including gender, age, height, weight, smoking status (never, ever/always), symptom (negative, positive chest symptoms), family history, the size, and location of lesion and the levels of serum tumor markers, including ferritin (FERR), squamous cell carcinoma antigen (SCCA), carbohydrate antigen 199 (CA 199), alpha-fetoprotein (AFP), carcinoembryonic antigen (CEA), cytokeratin 19 fragment antigen (CYFRA21-1), and neuron specific enolase (NSE) of each patient, were reviewed and recorded.

**Table 1:** Clinical and demographic characteristics of NSCLC patients

Characteristics	Total (*n* = 315)	SCC (*n* = 122)	ADC (*n* = 193)
Sex			
Male	200 (63.49)	109 (89.34)	91 (47.15)
Female	115 (36.51)	13 (10.66)	102 (52.85)
Age (mean ± SD, year)	61.89 ± 9.10	63.57 ± 8.31	60.82 ± 9.43
IASLC stage			
I A	71 (22.54)	13 (10.66)	58 (30.05)
I B	54 (17.14)	22 (18.03)	32 (16.58)
II A	24 (7.62)	12 (9.84)	12 (6.22)
II B	52 (16.51)	26 (21.31)	26 (13.47)
III A	114 (36.19)	49 (40.16)	65 (33.68)

Data in parentheses are percentages unless otherwise noted

*NSCLC* non-small cell lung cancer, *SCC* squamous cell carcinoma, *ADC* adenocarcinoma. *SD* standard deviation, *IASLC* International Association for the Study of Lung Cancer

### ^18^F-FDG PET/CT image acquisition and tumor segmentation

The ^18^F-FDG PET/CT scans were performed on a Biograph 16 PET/CT scanner (Siemens Healthcare, Erlangen, Germany) according to standard clinical scanning protocols. All patients fasted for at least 6 h before the scan, and none had a blood glucose levels > 8.7 mmol/L. A whole-body scan was acquired approximately 1 h after intravenous administering of 5.18 MBq/kg of ^18^F-FDG. The CT scans were performed first (120 kVp, 150 mAs, 0.33 s per rotation) using a slice thickness of 3.0 mm and reconstructed to a 512 × 512 matrix (voxel size: 0.98 × 0.98 × 3.0 mm^3^). Then, PET scans were performed with 2 min in each bed, a TrueX algorithm (2 iterations, 24 subsets, and 2 mm full width at half maximum) without filtering and smoothing was used to reconstruct the PET images. For all PET reconstructions, the matrix size was 200 × 200, resulting in anisotropic voxels of 4.07 × 4.07 × 3.0 mm^3^. The PET images were converted into SUV units by normalizing the activity concentration to the dosage of injected ^18^F-FDG and patient body weight.

Tumor segmentation was performed using Inveon Research Workplace (IRW, Siemens Healthcare, Erlangen, Germany) software. Two experienced nuclear medicine physicians drew boundaries in the axial, coronal, and sagittal PET scans that were large enough to include the primary tumor to delineate the VOIs using a threshold of 40% of SUV_max_ without knowing the pathology determined by consensus [21, 22]. To avoid the inclusion of areas with physiological ^18^F-FDG uptake within the regions of interest, a joint reading of both the CT and PET scans was performed side by side.

### Quantitative radiomic feature extraction

The radiomic features were extracted using a voxel-based methodology. First, the SUV values contained within the VOIs were relatively resampled to 64 different values to yield a limited range of values, with the goal of reducing the noise and normalizing the images [23]. Then, totally 212 radiomic features were automatically calculated and extracted from the PET and CT images for each lesion using the Chang Gung Image Texture Analysis (CGITA) that is compliant to the Image Biomarkers Standardization Initiative, which is an open-source software code with a graphical user interface for radiomics running on MATLAB (version 2019a, MathWorks Inc., Natick, MA) (supplementary data Fig. S1) [24]. The details of radiomic features were described in supplementary data (Table S1).

### Statistical analysis

The R (version 3.60, http://www.r-project.org) software was used for the statistical analysis. A comparison between the groups was performed using an independent *t* test or a Mann-Whitney *U* test for continuous variables and Fisher’s exact test or *χ*^2^ test for categorical variables. A two-sided *p* < 0.05 indicated statistical significance. Intra- and inter-class correlation coefficients (ICCs) were used to evaluate the consistency and reproducibility of the intra- and inter-observer agreements of the radiomic feature extractions. An ICC > 0.75 indicated good consistency.

### Features selection and prediction model establishment

Univariate analysis was applied to identify the relevant predictors of the NSCLC subtypes in the training set. Multivariate analysis was performed by the least absolute shrinkage and selection operator (LASSO) binary logistic regression with 10-fold cross-validation, which was used to select the most useful factors [25, 26]. The prediction models that were performed to differentiate ADC from SCC were developed by the linear fusion of the selected non-zero features weighted by their coefficients, with prediction scores (Pre-scores) of each model calculated for each patient.

### Prediction performance and clinical utility of prediction models

The performance of the models was evaluated by the receiver-operator characteristic curve (ROC) analysis and compared by the DeLong test. The AUC with 95% confidence interval (CI), sensitivity, specificity, and accuracy were calculated for each model. The clinical application value of the prediction models was determined and compared through the decision curve analysis (DCA) by quantifying the net benefit to the patient under different threshold probabilities in the queue.

### Development and validation of individualized nomogram

To provide a visually quantitative tool to predict the histologic subtypes for NSCLC patients, we developed a nomogram on the basis of the prediction model with the highest AUC and clinical utility in the training set [27]. Calibration curves were plotted to assess the calibration of the nomogram by bootstrapping (1000 bootstrap resamples) based on the internal (training set) and external (validation set) validity.

## Results

### Clinical characteristics and tumor markers of patients

In total, 315 NSCLC patients comprising 122 SCC patients and 193 ADC patients were eventually enrolled in this study. The patients’ clinical characteristics and tumor markers of training set are summarized and compared in Table 2, while ones of validation set are provided in supplementary data (Table S2). SCC patients were more likely to be elderly males who had taller heights, a history of smoking, obvious symptoms, and larger lesions, while ADC patients were more likely to be younger females who had never smoked, no obvious symptoms, and smaller lesions (*p* < 0.05). The levels of FERR, SCCA, CYFRA21-1, and NSE in SCC patients were higher than those in ADC patients (*p* < 0.05). There were no significant differences in patient’s weight, family history, lesion location, and levels of CA199, AFP, and CEA between the SCC and ADC groups according to the univariate analysis (*p* > 0.05).

**Table 2:** Comparison of clinical characteristics and tumor markers between SCC and ADC patients in training set

Characteristics	SCC (*n* = 80)	ADC (*n* = 140)	*p*
Sex			*< 0.001*
Male	72 (90.00)	69 (49.29)	
Female	8 (10.00)	71 (50.71)	
Age (year)	63.99 ± 8.99^#^	60.41 ± 9.65^#^	*0.007*
Height (m)	1.67 ± 0.08^#^	1.64 ± 0.08^#^	*0.002*
Weight (kg)	64.50 ± 10.06^#^	62.13 ± 10.29^#^	0.099
BMI	23.03 ± 3.00^#^	23.08 ± 3.15^#^	0.908
Smoking			*< 0.001*
Never	20 (25.00)	83 (59.29)	
Ever/Always	60 (75.00)	57 (40.71)	
Symptom			*< 0.001*
Negative	14 (17.50)	68 (48.57)	
Positive	66 (82.50)	72 (51.43)	
Family history			0.743
Negative	56 (70.00)	95 (67.86)	
Positive	24 (30.00)	45 (32.14)	
Location			0.939
Right lung	45 (56.25)	78 (55.71)	
Left lung	35 (43.75)	62 (44.29)	
Location_1			0.327
Upper lobe	43 (53.75)	84 (60.00)	
Middle lobe	6 (7.50)	11 (7.86)	
Lower lobe	31 (38.75)	45 (32.14)	
Size (cm)	5.56 ± 1.98^#^	3.69 ± 1.40^#^	*< 0.001*
FERR (ng/mL)	290.60 (190.60, 438.20)^*^	203.65 (124.53, 339.10)^*^	*0.001*
SCCA (ng/mL)	1.80 (1.30, 3.21)^*^	0.80 (0.50, 1.10)^*^	*0.001*
CA199 (U/mL)	13.18 (6.94, 23.26)^*^	10.19 (6.26, 18.66)^*^	0.344
AFP (ng/mL)	2.52 (1.92, 3.88)^*^	2.77 (2.19, 4.21)^*^	0.310
CEA (ng/mL)	3.38 (2.42, 4.88)^*^	3.88 (2.17, 8.52)^*^	0.483
CYFRA21-1 (ng/mL)	6.00 (4.58, 10.22)^*^	3.11 (2.32, 4.24)^*^	*< 0.001*
NSE (ng/mL)	12. 31 (10.75, 15.29)^*^	11.14 (9.93, 12.62)^*^	*< 0.001*

Data in parentheses are percentages unless otherwise noted

*BMI* body mass index, *FERR* ferritin, *SCCA* squamous cell carcinoma antigen, *CA* carbohydrate antigen, *AFP* alpha-fetoprotein, *CEA* carcinoembryonic antigen, *CYFRA21-1* cytokeratin 19 fragment antigen, *NSE* neuron specific enolase

^#^Values refer to mean ± standard deviation

*Values refer to median (interquartile range). *P* values were the results of univariate analysis of each characteristic, and the italics ones indicated statistical significance

### Features selection and prediction model establishment

A total of 315 regions with an increased ^18^F-FDG uptake were manually segmented, and 212 radiomic features were separately extracted by the two physicians. The agreement between the two physicians was excellent (all ICCs > 0.85, *p* < 0.05). Thus, the mean measurement values of the two physicians were used for further analysis.

**Fig. 2:**
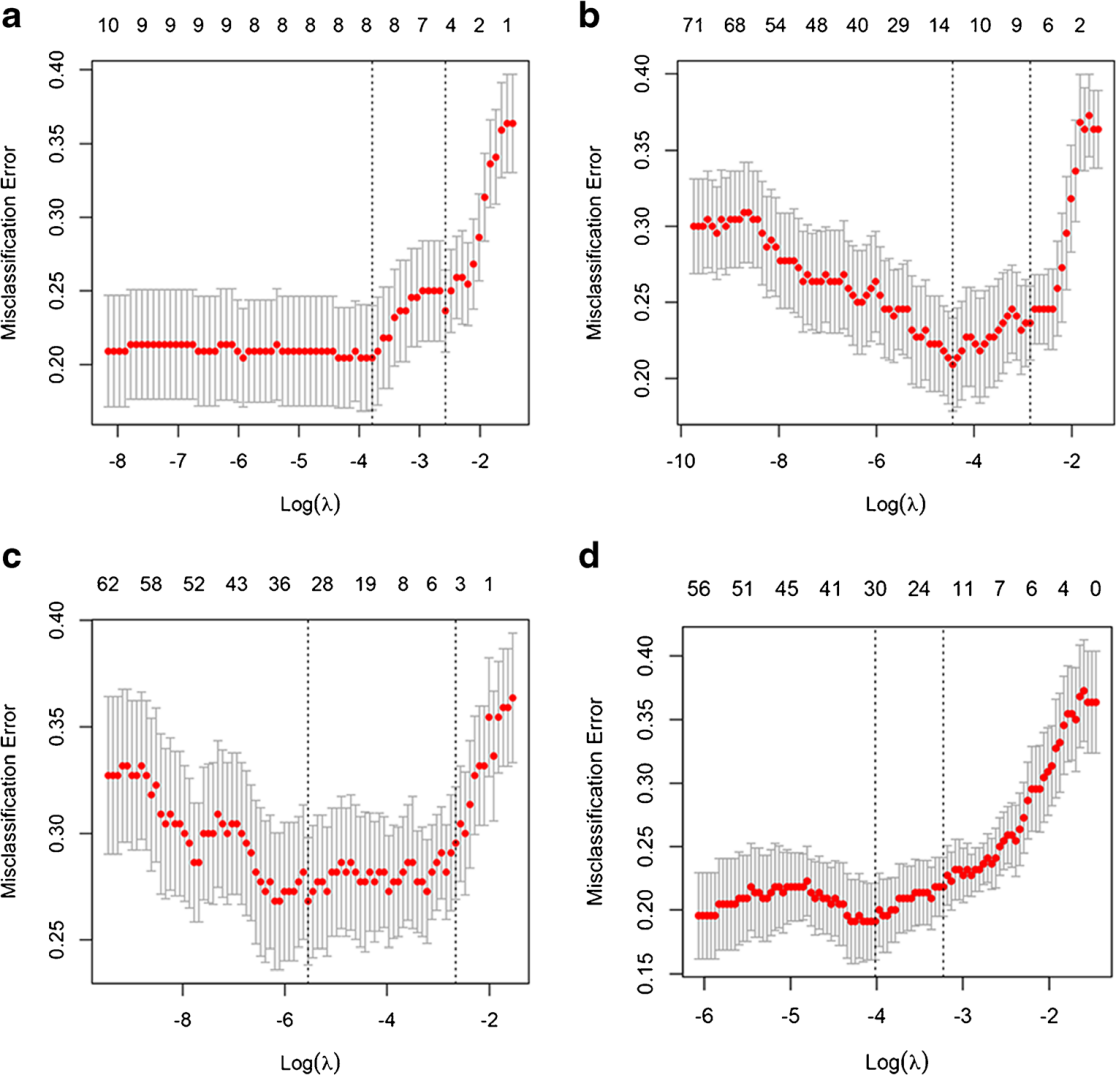
Features selection for prediction models using LASSO regression. The *X*-axis shows log (*λ*), and the *Y*-axis shows the model misclassification rate. The 5, 7, 3, and 14 features with non-zero coefficients are indicated for Clin-Lab Model (**a**), PET-Rad Model (**b**), CT-Rad Model (**c**), and Combined Model (**d**), respectively

For differentiating SCC from ADC, 4 independent prediction models (Clinical-Laboratory (Clin-Lab) Model, PET- Radiomic (Rad) Model, CT-Rad Model, and Combined Model) were built separately on the basis of selected clinical factors-tumor markers, PET radiomic parameters, CT radiomic parameters, and the combination of above features by LASSO regression in the training set (Fig. 2). The Pre-scores of each model for each patient were calculated using the following formulas:

$$ \mathrm{Pre}-\mathrm{score}\ \left(\mathrm{Clin}-\mathrm{Lab}\ \mathrm{Model}\right)=1.8145+0.8597\ast \mathrm{Sex}\ \left(\mathrm{Male}:0,\mathrm{Female}:1\right)-0.0847\ast \mathrm{Symptoms}\ \left(\mathrm{Negative}:0,\mathrm{Positive}:1\right)-0.3202\ast \mathrm{Size}\ \left(\mathrm{cm}\right)-0.0001\ast \mathrm{FERR}\ \left(\mathrm{ng}/\mathrm{mL}\right)-0.0020\ast \mathrm{SCCA}\ \left(\mathrm{ng}/\mathrm{mL}\right). $$

$$ \mathrm{Pre}-\mathrm{score}\ \left(\mathrm{PET}-\mathrm{Rad}\ \mathrm{Model}\right)=2.8790+1.4955\ast \mathrm{PET}\_{\mathrm{Coarseness}}^{\mathrm{Gray}\ \mathrm{Level}\ \mathrm{Neighborhood}\ \mathrm{Intensity}-\mathrm{difference}\ \mathrm{Matrix}\ \left(\mathrm{GLNIDM}\right)}+0.0025\ast \mathrm{PET}\_{\mathrm{Strength}}^{\mathrm{GLNIDM}}-0.0924\ast \mathrm{PET}\_\mathrm{Normalized}\_{\mathrm{Entropy}}^{\mathrm{Gray}\ \mathrm{Level}\ \mathrm{Co}-\mathrm{occurrence}\ \mathrm{Matrix}\ \left(\mathrm{GLCM}\right)}-0.4012\ast \mathrm{PET}\_{\mathrm{SUV}}_{\mathrm{min}}-0.1108\ast \mathrm{PET}\_{\mathrm{SUV}}_{\mathrm{mean}}-9.2039\ast \mathrm{PET}\_{\mathrm{Code}\ \mathrm{Similarity}}^{\mathrm{Texture}\ \mathrm{Feature}\ \mathrm{Coding}\ \mathrm{co}-\mathrm{occurrence}\ \mathrm{matrix}\ \left(\mathrm{TFCCM}\right)}+0.6994\ast \mathrm{PET}\_{\mathrm{Entropy}}^{\mathrm{Neighboring}\ \mathrm{Gray}\ \mathrm{Level}\ \mathrm{Dependence}\ \left(\mathrm{NGLD}\right)}. $$

$$ \mathrm{Pre}-\mathrm{score}\ \left(\mathrm{CT}-\mathrm{Rad}\ \mathrm{Model}\right)=1.7783\hbox{--} 0.0045\ast \mathrm{CT}\_\mathrm{Asphericity}-0.0024\ast \mathrm{CT}\_\mathrm{Entropy}\_\mathrm{prod}\_\mathrm{surface}\_\mathrm{area}+0.4938\ast \mathrm{CT}\_{\mathrm{Entropy}}^{\mathrm{NGLD}}. $$

$$ \mathrm{Pre}-\mathrm{score}\ \left(\mathrm{Combined}\ \mathrm{Model}\right)=3.6745+1.3360\ast \mathrm{Sex}\ \left(\mathrm{Male}:0,\mathrm{Female}:1\right)-0.2273\ast \mathrm{Size}\ \left(\mathrm{cm}\right)-0.0212\ast \mathrm{SCCA}\ \left(\mathrm{ng}/\mathrm{mL}\right)-0.0010\ast \mathrm{CYFRA}21.1\ \left(\mathrm{ng}/\mathrm{mL}\right)+0.0002\ast \mathrm{PET}\_{\mathrm{Strength}}^{\mathrm{GLNIDM}}+0.0001\ast \mathrm{PET}\_\mathrm{Normalized}\_{\mathrm{Contrast}}^{\mathrm{GLCM}}-0.0451\ast \mathrm{PET}\_\mathrm{Normalized}\_{\mathrm{Entropy}}^{\mathrm{GLCM}}-0.0498\ast \mathrm{PET}\_{\mathrm{SUV}}_{\mathrm{min}}-0.1138\ast \mathrm{PET}\_{\mathrm{SUV}}_{\mathrm{mean}}-0.0197\ast \mathrm{PET}\_\mathrm{Surface}\ {\mathrm{SUV}}_{\mathrm{mean}}\ 1+0.0469\ast \mathrm{PET}\_{\mathrm{Variance}}^{\mathrm{Texture}\ \mathrm{Feature}\ \mathrm{Coding}\ \left(\mathrm{TFC}\right)}-1.7315\ast \mathrm{CT}\_\mathrm{Second}\ {\mathrm{angular}\ \mathrm{moment}}^{\mathrm{TFCCM}}-0.4842\ast \mathrm{CT}\_{\mathrm{Correlation}}^{\mathrm{GLCM}}-0.0002\ast \mathrm{CT}\_\mathrm{Entropy}\_\mathrm{prod}\_\mathrm{surface}\_\mathrm{area}. $$

**Fig. 3:**
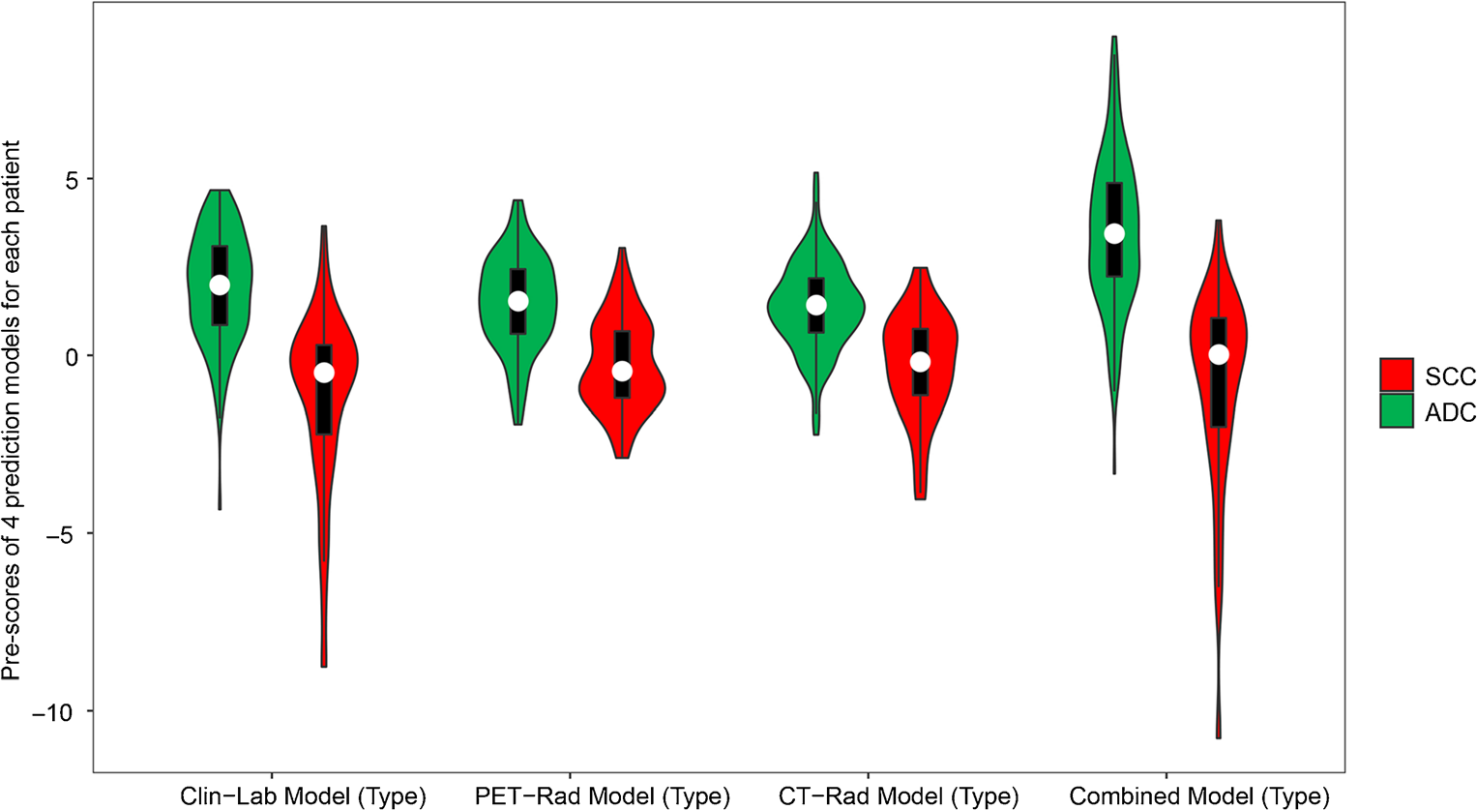
Violin plot of 4 prediction models for SCC and ADC patients in training set. The white dot represents the median. The black rectangle is the range from the lower quartile to the upper quartile. The black line running up and down through the violin diagram represents the range from the smallest non-outlier value to the largest non-outlier value

**Fig. 4:**
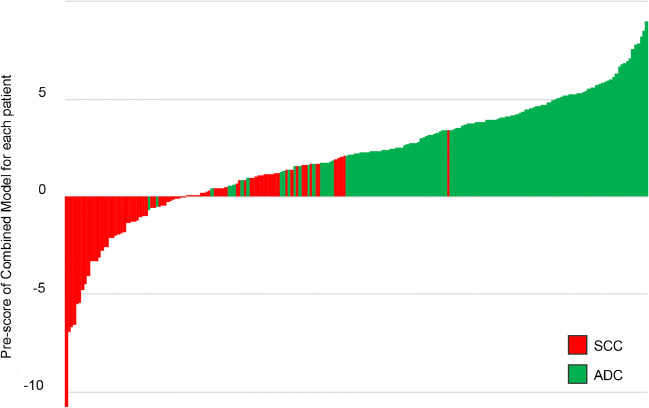
Pre-scores of the Combined Model for each patient in training set

ADC patients generally had higher Pre-scores in all prediction models than those in SCC patients (*p* < 0.001) (Figs. 3 and 4). The selected radiomic features of the prediction models between ADC and SCC patients are summarized and compared in supplementary data (Table S3).

### Prediction performance and clinical utility of prediction models

**Fig. 5:**
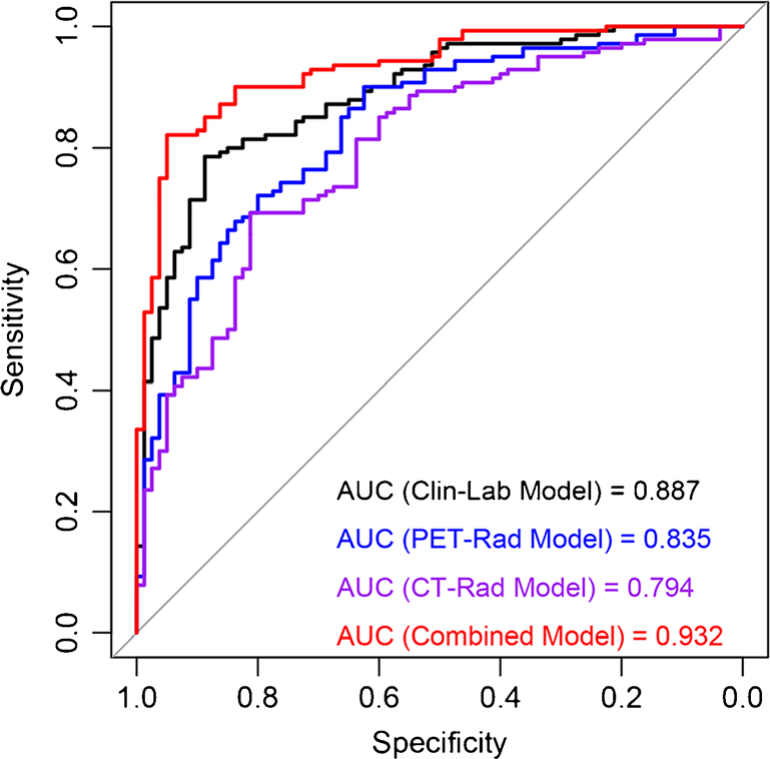
Receiver-operating characteristic analysis of prediction models for predicting NSCLC subtypes in training set

The performance of these 4 prediction models to discriminate SCC from ADC is shown in Fig. 5. The Clin-Lab Model consisted of 3 clinical factors and 2 tumor markers, the PET-Rad Model consisted of 7 PET radiomic parameters, and the CT-Rad Model consisted of 3 CT radiomic parameters that were all significantly associated with the NSCLC pathological subtypes (AUCs (training set) = 0.887, 0.835, 0.784; AUCs (validation set) = 0.860, 0.740, 0.710, respectively).

The DeLong test showed that the Combined Model, which consisted of 2 clinical factors, 2 tumor markers, 7 PET radiomic parameters, and 3 CT radiomic parameters, presented the optimal discrimination and best predictive sensitivity, specificity, and accuracy among the 4 models in both the training set (AUC (95% CI) = 0.932 (0.900–0.964), sensitivity = 96.25%, specificity = 95.00%, accuracy = 84.09%) and validation set (AUC (95% CI) = 0.901 (0.840–0.957), sensitivity = 93.55%, specificity = 81.25%, accuracy = 85.95%) (both *p* < 0.05) **(**Table 3**)**.

**Table 3:** Performance of prediction models for predicting subtypes in NSCLC

**Training set**	**AUC (95% CI)**	**Sen (%)**	**Spe (%)**	**Acc (%)**
Clin-Lab Model	0.887 (0.843–0.931)	78.57	88.75	80.91
PET-Rad Model	0.835 (0.780–0.890)	90.00	62.50	78.64
CT-Rad Model	0.784 (0.733–0.855)	69.29	81.25	75.00
Combined Model	0.932 (0.900–0.964)	96.25	95.00	84.09
**Validation set**	**AUC (95% CI)**	**Sen (%)**	**Spe (%)**	**Acc (%)**
Clin-Lab Model	0.860 (0.789–0.931)	80.65	76.56	72.63
PET-Rad Model	0.740 (0.639–0.840)	83.87	75.00	66.32
CT-Rad Model	0.710 (0.606–0.815)	70.97	60.94	68.42
Combined Model	0.901 (0.840–0.957)	93.55	81.25	85.95

*Clin-Lab* Clinical-Laboratory, *PET-Rad* positron emission tomography-radiomics, *CT-Rad* computed tomography-radiomics, *AUC* area under the receiver operating curve, *CI* confidence interval, *Sen* sensitivity, *Spe* specificity, *Acc* accuracy

**Fig. 6:**
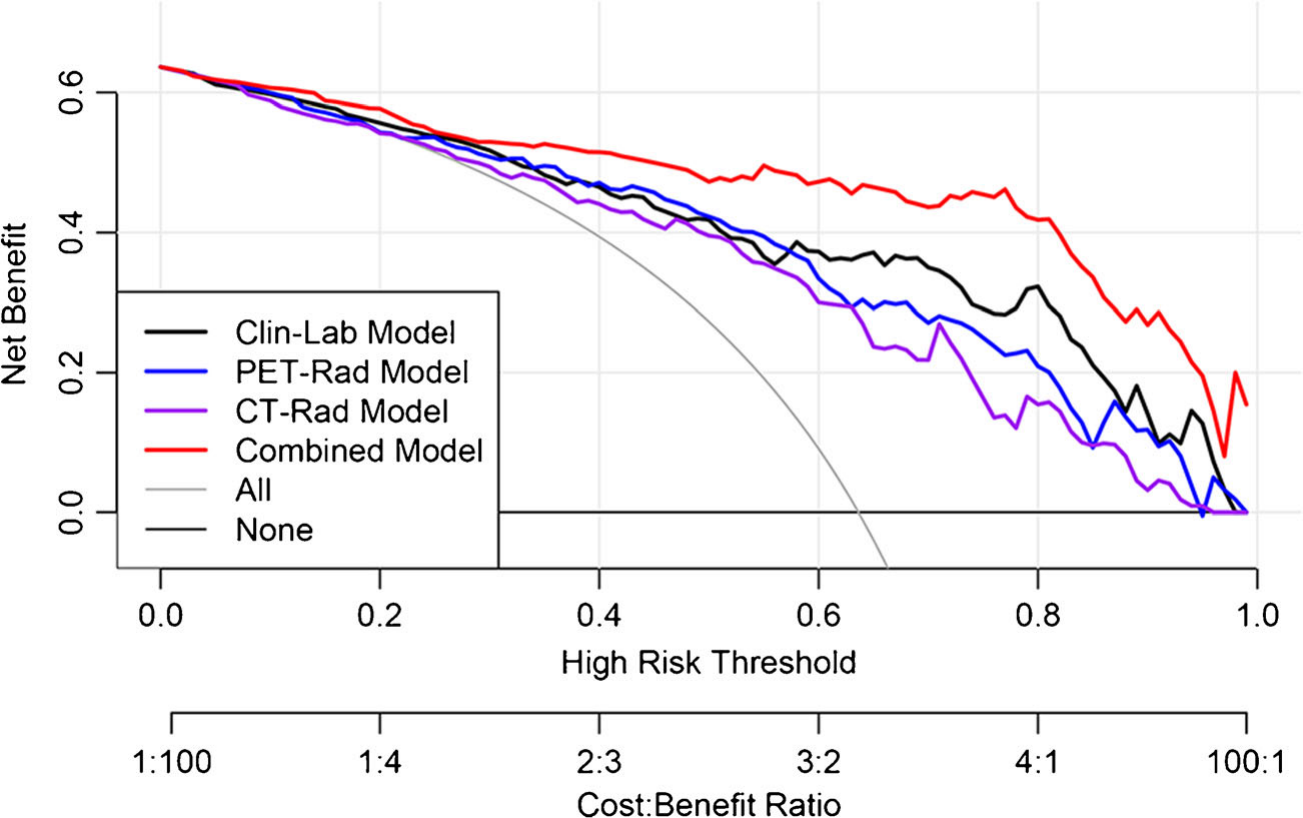
Decision curve analysis (DCA) of prediction models in training set. The *X*-axis represented the threshold probability that was where the expected benefit of treatment was equal to the expected benefit of avoiding treatment. The *Y*-axis represented the net benefit. The gray and black line represented the hypothesis that all NSCLC patients were ADC and SCC, respectively

The DCA also showed that the Combined Model was the most reliable clinical treatment tool for predicting the histologic subtypes in NSCLC when the threshold probability was greater than 10% (Fig. 6).

### Development and validation of individualized nomogram

**Fig. 7:**
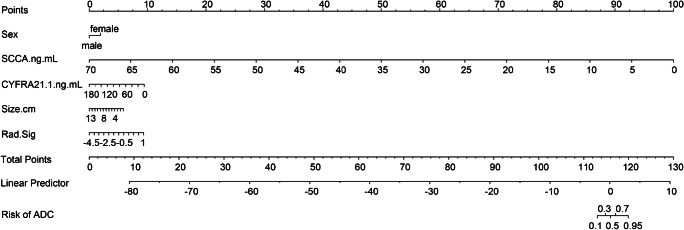
Developed the prediction nomogram based on Combined Model in training set. The probability of each predictor could be converted into scores according to the first scale “Points” at the top of the nomogram. After adding up the corresponding prediction probability at the bottom of the nomogram was the risk of ADC

**Fig. 8:**
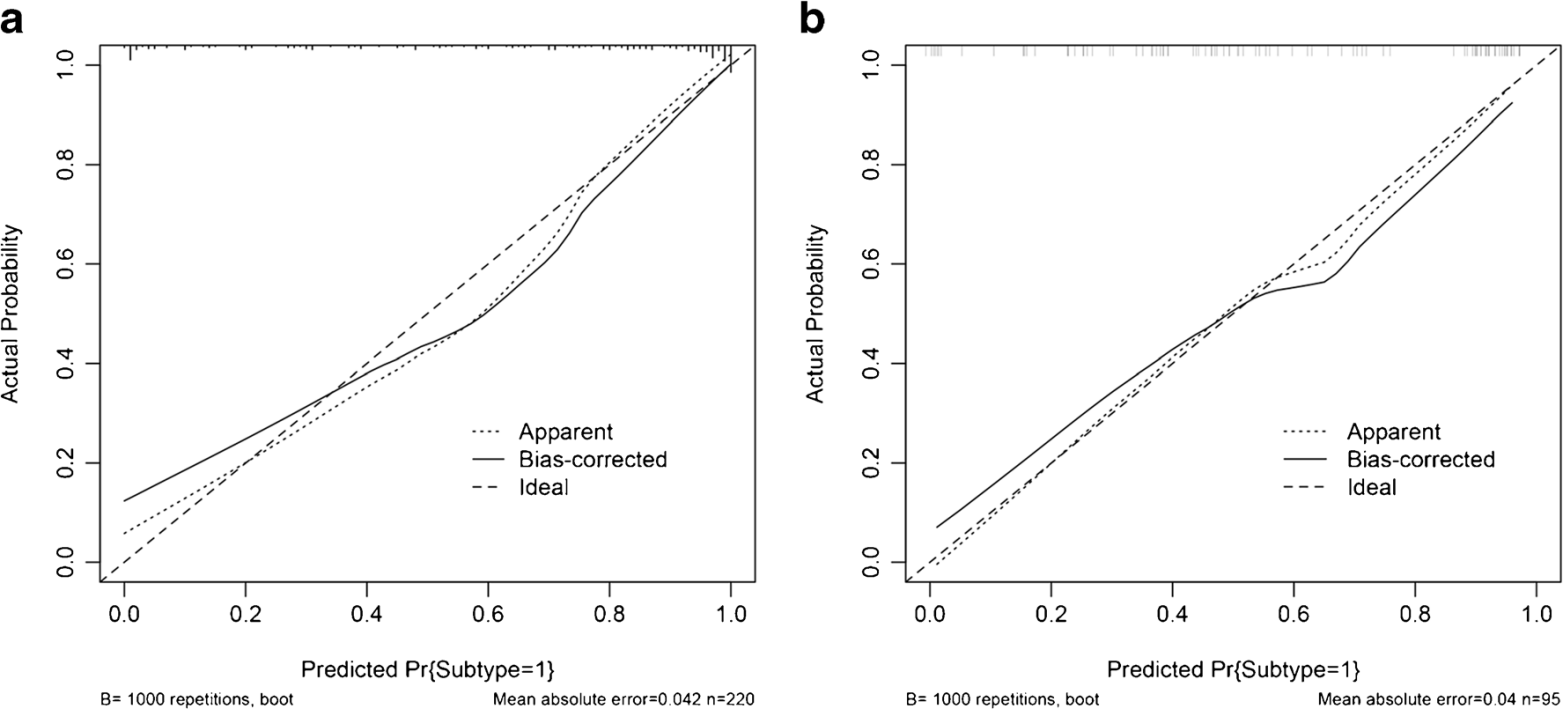
Calibration curves of nomogram in training (**a**) and validation (**b**) sets, respectively. The *X*-axis represented the predicted probability estimated by nomogram, whereas the *Y*-axis represented the actual observed rates. The solid line represented the ideal reference line that predicted NSCLC subtypes corresponds to the actual outcome, the short-dashed line represented the apparent prediction of nomogram, and the long-dashed line represented the ideal estimation. Calibration curves showed the actual probability corresponded closely to the prediction of nomogram

According to the above results, we generated an individualized nomogram based on the Combined Model’s risk factors for the visualization (Fig. 7). The calibration curves of the nomogram for the probability of ADC demonstrated a good agreement between the prediction by the nomogram and the actual observation in both the training and validation sets (Fig. 8).

## Discussion

In this study, we successfully constructed and validated a Combined Model containing clinical factors, tumor markers, and radiomic features extracted from both the PET and CT images, which held an excellent performance in noninvasively stratifying NSCLC patients according to their pathological subtypes. In addition, we developed a visually quantitative nomogram for conveniently using this prediction model in clinical practice.

Of the clinical factors selected in the Combined Model, sex differences among NSCLC patients have been widely reported, with that SCC affecting more males than females [28]. The lesions are generally bigger in SCC patients than in ADC patients [29]. Tumor markers in serum are beneficial for the diagnosis and prognosis of NSCLC [30]. The serum levels of SCCA and CYFRA21-1 are highly sensitive in NSCLC and significantly higher in SCC than in ADC [31]. The results of this study are consistent with the conclusions of the above reports.

Different pathological subtypes lead to various clinical strategies and prognoses for NSCLC patients [5, 32]. The PET/CT-based radiomic is a relatively new quantitative imaging technique for the noninvasive assessment of tumors [33]. Ha S, et al. found that PET radiomic features were significantly different between ADC and SCC with 0.90 linear separability, but the study population was only 30 people [34]. Koyasu S, et al. also showed that PET radiomics was indeed useful in NSCLC subtypes with an AUC of 0.843 [15]. However, the radiomic approaches in the above studies were not be validated in another independent dataset. In this study, both the PET and CT radiomic approaches were applied and validated to have a good performance in the classification of NSCLC subtypes (AUCs (PET-Rad Model and CT-Rad Model) = 0.835 and 0.784, respectively). The above results indicated that the relationship between medical images and tumor molecular phenotypes can be established by radiomics, and then the diagnostic information of tumors can be obtained noninvasively through medical images for some patients who are not eligible for biopsy.

In addition, since radiomic extracts information from the tumor, an appropriate tumor segmentation algorithm is important for measuring tumor image parameters [35]. Ideally, the chosen segmentation method is both accurate and robust. Bashir et al. had compared various segmentation algorithms (freehand, 40% of maximum intensity threshold, and fuzzy locally adaptive Bayesian algorithms) in terms of inter-observer reproducibility and prognostic capability of texture parameters derived from NSCLC ^18^F-FDG PET/CT images [21]. They found that the models generated by all three segmentation algorithms were of at least equivalent utility. Moreover, segmentation with 40% of maximum threshold leads to the best reproducibility of image biomarkers when used by different observers. In this study, the agreements of the radiomic feature extraction using semiautomatic threshold-based methods were excellent (all ICCs > 0.85, *p* < 0.05). The high ability to reproduce and validate radiomic studies is vital to generating sufficient and convincing scientific evidence for translating potential applications into clinical practice [33, 36].

This study also explored whether the prediction performance based on radiomics could be further improved by combining with clinical factors and tumor marker levels. The Combined Model established in the present study not only significantly improved the prediction efficiency for subtype compared to these factors alone in both the training and validation sets (AUCs = 0.932 (training set), 0.901 (validation set), respectively) but also had higher performance than previous researches [14–17]. This discrepancy may be related to the complete and standard preoperative baseline data and postoperative pathological reports from a single center, as well as the appropriate algorithm [37]. The results of this study confirm the hypothesis and indicate that the heterogeneity of tumors can be evaluated more comprehensively by combining with multiscale characteristics of tumors, consistent with the report [38].

In addition, we generated an integrated nomogram on the basis of the Combined Model for facilitating its use in clinical practice. Clinical factors such as patient’s sex and age are recorded routinely at hospital admission. Moreover, we strongly recommend that serum tumor marker levels should be evaluated in patients who are highly suspected of having NSCLC or initially diagnosed with NSCLC, especially SCCA, CYFRA21-1. Both physicians and patients could perform a preoperative individualized prediction of the risk of ADC with this easy-to-use scoring tool, which can provide a noninvasive and accurate approach for patients who are unwilling or unable to undergo biopsy to develop more reasonable and effective treatment plans, especially the need of targeted therapy [39]. The DCA showed that if the threshold probability of a patient or doctor is > 10%, using this nomogram to predict the subtype adds more benefit than either the treat-all-patients as SCC or the treat-all-patients as ADC, which is more valuable for the current trend toward personalized medicine [40].

Although the results were encouraging, the present study had several limitations. Firstly, the sample selection was biased in this single-center retrospective study, and a new multicenter prospective study is still necessary to be designed for the further evaluation and verification of the generalization ability of the models. Secondly, some NSCLC patients, especially ADC patients, were excluded from the radiomic analysis due to the faint ^18^F-FDG uptake or small tumor size to ensure the quality of images and textural data. Small lesions are easier to be discovered in the early stage with the increasing use of imaging screening for lung cancer. Thus, a more sensitive tool that adaptively detects small tumors will be an important direction for future work. Finally, the patients with non-primary lung lesions were also excluded due to the purpose of this study. Noticeably that both primary and metastatic pulmonary nodules are very important for patients and clinical settings in the cancer center. The prediction model that widely used for lung lesions will be continually explored in future studies.

In conclusion, an integrated nomogram was constructed and validated in our study, which could provide a relatively accurate, convenient, and noninvasive method for the individualized discrimination between ADC and SCC in NSCLC patients, assisting in clinical decision making for precision treatment.

## Electronic supplementary material

ESM 1 (DOCX 790 kb)

## Data Availability

Yes

## Abbreviations

ADC: adenocarcinoma
AFP: alpha-fetoprotein
ALK: anaplastic lymphoma kinase
AUC: area under curve
BMI: body mass index
CA: carbohydrate antigen
CEA: carcinoembryonic antigen
CI: confidence interval
Clin-Lab: Clinical-Laboratory
CYFRA21-1: cytokeratin 19 fragment antigen
DCA: decision curve analysis
EGFR: epidermal growth factor receptor
FERR: ferritin
FDG: fluorodeoxyglucose
GLCM: Gray Level Co-occurrence Matrix
GLNIDM: Gray Level Neighborhood Intensity-difference Matrix
ICC: intra- and inter-class correlation coefficient
LASSO: least absolute shrinkage and selection operator
NGLD: Neighboring Gray Level Dependence
NSCLC: non-small cell lung cancer
NSE: neuron specific enolase
PET/CT: positron emission tomography/computed tomography
Pre-score: prediction score
Rad: radiomics
ROC: receiver operating characteristic
SCC: squamous cell carcinoma
SCCA: squamous cell carcinoma antigen
SD: standard deviation
SUV: standardized uptake value
TFC: Texture Feature Coding
TFCCM: Texture Feature Coding co-occurrence matrix
VOI: volume of interest
WHO: World Health Organization

## References

[1] Siegel RL, Miller KD, Jemal A (2019). Cancer statistics, 2019. CA-Cancer J Clin.

[2] Herbst RS, Morgensztern D, Boshoff C (2018). The biology and management of non-small cell lung cancer. Nature..

[3] Thomas A, Liu SV, Subramaniam DS, Giaccone G (2015). Refining the treatment of NSCLC according to histological and molecular subtypes. Nat Rev Clin Oncol.

[4] Detterbeck FC, Boffa DJ, Kim AW, Tanoue LT (2017). The eighth edition lung cancer stage classification. Chest..

[5] Yuan CZ, Tao XT, Zheng DF, Pan YJ, Ye T, Hu H (2019). The lymph node status and histologic subtypes influenced the effect of postoperative radiotherapy on patients with N2 positive IIIA non-small cell lung cancer. J Surg Oncol.

[6] Cooper WA, O'Toole S, Boyer M, Horvath L, Mahar A (2011). What’s new in non-small cell lung cancer for pathologists: the importance of accurate subtyping, EGFR mutations and ALK rearrangements. Pathology..

[7] Sutiman N, Weng S, Tan EH, Lim WT, Kanesvaran R, Ng QS (2017). EGFR mutation subtypes influence survival outcomes’ following first-line gefitinib therapy in advanced Asian NSCLC patients. J Thorac Oncol.

[8] Ebrahimi M, Auger M, Jung S, Fraser RS (2016). Diagnostic concordance of non-small cell lung carcinoma subtypes between biopsy and cytology specimens obtained during the same procedure. Cancer Cytopathol.

[9] Manhire A, Charig M, Clelland C, Gleeson F, Miller R, Moss H (2003). Guidelines for radiologically guided lung biopsy. Thorax..

[10] de Margerie-Mellon C, de Bazelaire C, de Kerviler E (2016). Image-guided biopsy in primary lung cancer: why, when and how. Diagn Interv Imaging.

[11] Osmani L, Askin F, Gabrielson E, Li QK (2018). Current WHO guidelines and the critical role of immunohistochemical markers in the subclassification of non-small cell lung carcinoma (NSCLC): moving from targeted therapy to immunotherapy. Semin Cancer Biol.

[12] Lambin P, Rios-Velazquez E, Leijenaar R, Carvalho S, van Stiphout RG, Granton P (2012). Radiomics: extracting more information from medical images using advanced feature analysis. Eur J Cancer.

[13] Huang YQ, Liang CH, He L, Tian J, Liang CS, Chen X et al. Development and validation of a radiomics nomogram for preoperative prediction of lymph node metastasis in colorectal cancer. J Clin Oncol. 2016;34(18):2157−+. 10.1200/JCO.2015.65.9128. 10.1200/JCO.2015.65.9128 27138577

[14] Ma Y, Feng W, Wu ZY, Liu MY, Zhang F, Liang ZG et al. Intra-tumoural heterogeneity characterization through texture and colour analysis for differentiation of non-small cell lung carcinoma subtypes. Phys Med Biol. 2018;63(16). 10.1088/1361-6560/aad648. 10.1088/1361-6560/aad648 30051884

[15] Koyasu S, Nishio M, Isoda H, Nakamoto Y, Togashi K (2020). Usefulness of gradient tree boosting for predicting histological subtype and EGFR mutation status of non-small cell lung cancer on F-18 FDG-PET/CT. Ann Nucl Med.

[16] Hyun SH, Ahn MS, Koh YW, Lee SJ (2019). A machine-learning approach using PET-based radiomics to predict the histological subtypes of lung cancer. Clin Nucl Med.

[17] Sha X, Gong GZ, Qiu QT, Duan JH, Li DW, Yin Y. Identifying pathological subtypes of non-small-cell lung cancer by using the radiomic features of F-18-fluorodeoxyglucose positron emission computed tomography. Transl Cancer Res. 2019;8(5):1741−+. 10.21037/tcr.2019.08.20. 10.21037/tcr.2019.08.20 PMC8799142 35116924

[18] Dagogo-Jack I, Shaw AT (2018). Tumour heterogeneity and resistance to cancer therapies. Nat Rev Clin Oncol.

[19] Sikaroodi M, Galachiantz Y, Baranova A (2010). Tumor markers: the potential of “omics” approach. Curr Mol Med.

[20] Travis WD, Brambilla E, Nicholson AG, Yatabe Y, Austin JHM, Beasley MB (2015). The 2015 World Health Organization classification of lung tumors impact of genetic, clinical and radiologic advances since the 2004 classification. J Thorac Oncol.

[21] Bashir U, Azad G, Siddique MM, Dhillon S, Patel N, Bassett P, et al. The effects of segmentation algorithms on the measurement of F-18-FDG PET texture parameters in non-small cell lung cancer. EJNMMI Res. 2017;7. 10.1186/s13550-017-0310-3. 10.1186/s13550-017-0310-3 PMC5529305 28748524

[22] Cook GJR, Azad G, Owczarczyk K, Siddique M, Goh V (2018). Challenges and promises of PET radiomics. Int J Radiat Oncol Biol Phys.

[23] Yang ZY, Sun YF, Xu XP, Zhang YP, Zhang JP, Xue J (2017). The assessment of estrogen receptor status and its intratumoral heterogeneity in patients with breast cancer by using 18F-fluoroestradiol PET/CT. Clin Nucl Med.

[24] Fang YH, Lin CY, Shih MJ, Wang HM, Ho TY, Liao CT (2014). Development and evaluation of an open-source software package “CGITA” for quantifying tumor heterogeneity with molecular images. Biomed Res Int.

[25] Tibshirani R (1996). Regression shrinkage and selection via the Lasso. J R Stat Soc Ser B-Methodol.

[26] Sauerbrei W, Royston P, Binder H (2007). Selection of important variables and determination of functional form for continuous predictors in multivariable model building. Stat Med.

[27] Balachandran VP, Gonen M, Smith JJ, DeMatteo RP (2015). Nomograms in oncology: more than meets the eye. Lancet Oncol.

[28] Caldarella A, Crocetti E, Comin CE, Janni A, Pegna AL, Paci E (2007). Gender differences in non-small cell lung cancer: a population-based study. Ejso..

[29] Li M, Wu N, Zheng R, Liang Y, Liu Y, Zhang W (2013). Primary tumor PET/CT [(1)(8)F]FDG uptake is an independent predictive factor for regional lymph node metastasis in patients with non-small cell lung cancer. Cancer Imaging.

[30] Yang GJ, Xiao ZQ, Tang CL, Deng Y, Huang H, He ZY. Recent advances in biosensor for detection of lung cancer biomarkers. Biosens Bioelectron. 2019;141. 10.1016/j.bios.2019.111416. 10.1016/j.bios.2019.111416 31279179

[31] Liu LJ, Teng JL, Zhang LJ, Cong PS, Yao Y, Sun GR, et al. The combination of the tumor markers suggests the histological diagnosis of lung cancer. Biomed Res Int. 2017. 10.1155/2017/2013989. 10.1155/2017/2013989 PMC5451759 28607926

[32] McAleese J, Taylor A, Walls GM, Hanna GG (2019). Differential relapse patterns for non-small cell lung cancer subtypes adenocarcinoma and squamous cell carcinoma: implications for radiation oncology. Clin Oncol.

[33] Zwanenburg A (2019). Radiomics in nuclear medicine: robustness, reproducibility, standardization, and how to avoid data analysis traps and replication crisis. Eur J Nucl Med Mol Imaging.

[34] Ha S, Choi H, Cheon GJ, Kang KW, Chung JK, Kim EE (2014). Autoclustering of non-small cell lung carcinoma subtypes on (18)F-FDG PET using texture analysis: a preliminary result. Nucl Med Mol Imaging.

[35] Kumar V, Gu YH, Basu S, Berglund A, Eschrich SA, Schabath MB (2012). Radiomics: the process and the challenges. Magn Reson Imaging.

[36] Park JE, Park SY, Kim HJ, Kim HS (2019). Reproducibility and generalizability in radiomics modeling: possible strategies in radiologic and statistical perspectives. Korean J Radiol.

[37] Yin P, Mao N, Zhao C, Wu JF, Sun C, Chen L (2019). Comparison of radiomics machine-learning classifiers and feature selection for differentiation of sacral chordoma and sacral giant cell tumour based on 3D computed tomography features. Eur Radiol.

[38] Lv WB, Yuan QY, Wang QS, Ma JH, Feng QJ, Chen WF (2019). Radiomics analysis of PET and CT components of PET/CT imaging integrated with clinical parameters: application to prognosis for nasopharyngeal carcinoma. Mol Imaging Biol.

[39] Salem A, Asselin MC, Reymen B, Jackson A, Lambin P, West CML et al. Targeting hypoxia to improve non-small cell lung cancer outcome. JNCI-J Natl Cancer Inst. 2018;110(1). 10.1093/jnci/djx160. 10.1093/jnci/djx160 28922791

[40] Rocco G, Morabito A, Leone A, Muto P, Fiore F, Budillon A (2016). Management of non-small cell lung cancer in the era of personalized medicine. Int J Biochem Cell Biol.

