# Supplementary material for: Machine learning based on clinico-biological features integrated 18F-FDG PET/CT radiomics for distinguishing squamous cell carcinoma from adenocarcinoma of lung
Source: Eur J Nucl Med Mol Imaging. 2020 Oct 15;48(5):1538–49. doi: 10.1007/s00259-020-05065-6 (PMC8113203; doi:10.1007/s00259-020-05065-6)
Supplement: Supplementary file 1 — (DOCX 790 kb) [file 259_2020_5065_MOESM1_ESM.docx]

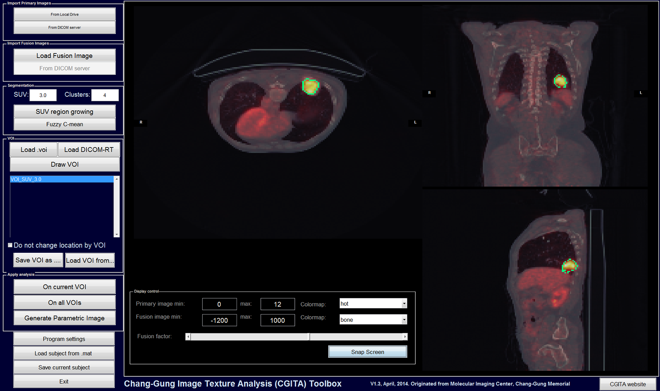


**Fig. S1** Tumor segmentation was performed using IRW Siemens software (Inveon Research Workstation, Germany). For parameter calculation, CGITA, developed by Fang et al., was used.

**Table S1.** Specific categories of radiomics features

| Matrix | Radiomics Feature Name | Abbreviations |
| --- | --- | --- |
| Gray Level Co-occurrence Matrix (GLCM) | Second Angular Moment | SAM^GLCM^ |
|  | Contrast | Contrast^GLCM^ |
|  | Entropy | Entropy^GLCM^ |
|  | Homogeneity | Homogeneity^GLCM^ |
|  | Dissimilarity | Dissimilarity^GLCM^ |
|  | Inverse difference moment | IDM^GLCM^ |
|  | Correlation | Correlation^GLCM^ |
| Normalized (NL) GLCM | Normalized Second Angular Moment | NL_SAM^GLCM^ |
|  | Normalized Contrast | NL_Contrast^GLCM^ |
|  | Normalized Entropy | NL_Entropy^GLCM^ |
|  | Normalized Homogeneity | NL_Homogeneity^GLCM^ |
|  | Normalized Dissimilarity | NL_Dissimilarity^GLCM^ |
|  | Normalized Inverse difference moment | NL_IDM^GLCM^ |
| Gray Level Run-length Matrix (GLRM) | Short run emphasis | SRE^GLRM^ |
|  | Long run emphasis | LRE^GLRM^ |
|  | Intensity variability | IV^GLRM^ |
|  | Run-length variability | RLV^GLRM^ |
|  | Run percentage | RP^GLRM^ |
|  | Low-intensity run emphasis | LIRE^GLRM^ |
|  | High-intensity run emphasis | HIRE^GLRM^ |
|  | Low-intensity short-run emphasis | LISRE^GLRM^ |
|  | High-intensity short-run emphasis | HISRE^GLRM^ |
|  | Low-intensity long-run emphasis | LILRE^GLRM^ |
|  | High-intensity long-run emphasis | HILRE^GLRM^ |
| Gray Level Neighborhood Intensity-difference Matrix (GLNIDM) | Coarseness | Coarseness^GLNIDM^ |
|  | Contrast | Contrast^GLNIDM^ |
|  | Busyness | Busyness^GLNIDM^ |
|  | Complexity | Complexity^GLNIDM^ |
|  | Strength | Strength^GLNIDM^ |
| Gray Level Size Zone Matrix (GLSZM) | Short-zone emphasis | SZE^GLSZM^ |
|  | Large-zone emphasis | LZE^GLSZM^ |
|  | Intensity variability | IV^GLSZM^ |
|  | Size-zone variability | SZV^GLSZM^ |
|  | Zone percentage | ZP^GLSZM^ |
|  | Low-intensity zone emphasis | LIZE^GLSZM^ |
|  | High-intensity zone emphasis | HIZE^GLSZM^ |
|  | Low-intensity short-zone emphasis | LISZE^GLSZM^ |
|  | High-intensity short-zone emphasis | HISZE^GLSZM^ |
|  | Low-intensity large-zone emphasis | LILZE^GLSZM^ |
|  | High-intensity large-zone emphasis | HILZE^GLSZM^ |
| Standardized Uptake Value (SUV) Statistics | Minimum SUV | SUV_min_ |
|  | Maximum SUV | SUV_max_ |
|  | Mean SUV | SUV_mean_ |
|  | SUV Variance | Variance |
|  | SUV SD | SD |
|  | Coefficient of variance | CV |
|  | SUV Skewness | Skewness |
|  | SUV Kurtosis | Kurtosis |
|  | SUV bias-corrected Skewness | Skewness_bias-corrected_ |
|  | SUV bias-corrected Kurtosis | Kurtosis_bias-corrected_ |
|  | Total legion glycolysis | TLG |
|  | Metabolic tumor volume | MTV |
|  | Entropy | Entropy |
|  | SUL_peak_ | SUL_peak_ |
|  | Surface area | Surface area |
|  | Asphericity | Asphericity |
|  | Asphericity 2 | Asphericity 2 |
|  | Asphericity 3 | Asphericity 3 |
|  | Surface mean SUV 1 | Surface SUV_mean_ 1 |
|  | Surface total SUV 1 | Surface total 1 |
|  | Surface SUV entropy 1 | Surface entropy 1 |
|  | Surface SUV variance 1 | Surface variance 1 |
|  | Surface SUV SD 1 | Surface SD 1 |
|  | Surface SUV NSR 1 | Surface NSR 1 |
|  | Surface mean SUV 2 | Surface SUV_mean_ 2 |
|  | Surface total SUV 2 | Surface total 2 |
|  | Surface SUV entropy 2 | Surface entropy 2 |
|  | Surface SUV variance 2 | Surface variance 2 |
|  | Surface SUV SD 2 | Surface SD 2 |
|  | Surface SUV NSR 2 | Surface NSR 2 |
|  | Surface mean SUV 3 | Surface SUV_mean_ 3 |
|  | Surface total SUV 3 | Surface total 3 |
|  | Surface SUV entropy 3 | Surface entropy 3 |
|  | Surface SUV variance 3 | Surface variance 3 |
|  | Surface SUV SD 3 | Surface SD 3 |
|  | Surface SUV NSR 3 | Surface NSR 3 |
|  | Surface mean SUV 4 | Surface SUV_mean_ 4 |
|  | Surface total SUV 4 | Surface total 4 |
|  | Surface SUV entropy 4 | Surface entropy 4 |
|  | Surface SUV variance 4 | Surface variance 4 |
|  | Surface SUV SD 4 | Surface SD 4 |
|  | Surface SUV NSR 4 | Surface NSR 4 |
|  | SUVmean_prod_asphericity | SUVmean_prod_A |
|  | SUVmax_prod_asphericity | SUVmax_prod_A |
|  | Entropy_prod_asphericity | Entropy_prod_A |
|  | SULpeak_prod_asphericity | SULpeak_prod_A |
|  | SUVmean_prod_surface_area | SUVmean_prod_SA |
|  | SUVmax_prod_surface_area | SUVmax_prod_SA |
|  | Entropy_prod_surface_area | Entropy_prod_SA |
|  | SULpeak_prod_surface_area | SULpeak_prod_SA |
| Texture Spectrum | Max spectrum | Spectrum_max_^TS^ |
| Texture Feature Coding (TFC) | Coarseness | Coarneness^TFC^ |
|  | Mean convergence | MC^TFC^ |
|  | Variance | Variance^TFC^ |
| Texture Feature Coding co-occurrence matrix (TFCCM) | Second angular moment | SAM^TFCCM^ |
|  | Contrast | Contrast^TFCCM^ |
|  | Entropy | Entropy^TFCCM^ |
|  | Homogeneity | Homogeneity^TFCCM^ |
|  | Intensity | Intensity^TFCCM^ |
|  | Inverse difference moment | IDM^TFCCM^ |
|  | Code Entropy | CE^TFCCM^ |
|  | Code Similarity | CS^TFCCM^ |
| Neighboring Gray Level Dependence (NGLD) | Small number emphasis | SNE^NGLD^ |
|  | Large number emphasis | LNE^NGLD^ |
|  | Number nonuniformity | NNU^NGLD^ |
|  | Second moment | SM^NGLD^ |
|  | Entropy | Entropy^NGLD^ |

**Table S2** Comparison of clinical characteristics and tumor markers between SCC and ADC patients in validation set

| Characteristics | SCC (n=42) | ADC (n=53) | *p* |
| --- | --- | --- | --- |
| Sex |  |  | **<0.001** |
| Male | 37 (88.10) | 22 (41.51) |  |
| Female | 5 (11.90) | 31 (58.49) |  |
| Age (y) | 62.79 ± 6.86 ^#^ | 61.91 ± 9.83 ^#^ | 0.597 |
| Height (m) | 1.67 ± 0.07 ^#^ | 1.63 ± 0.09 ^#^ | **0.018** |
| Weight (Kg) | 63.88 ± 10.61 ^#^ | 61.08 ± 9.83 ^#^ | 0.186 |
| BMI | 22.96 ± 3.38 ^#^ | 20.01 ± 2.51 ^#^ | 0.935 |
| Smoking |  |  | **<0.001** |
| Never | 7 (16.67) | 29 (54.72) |  |
| Ever/Always | 35 (83.33) | 24 (45.28) |  |
| Symptom |  |  | 0.087 |
| Negative | 14 (33.33) | 27 (50.94) |  |
| Positive | 28 (66.67) | 26 (49.06) |  |
| Family History |  |  | 0.475 |
| Negative | 35 (83.33) | 41 (77.36) |  |
| Positive | 7 (16.67) | 12 (22.64) |  |
| Location |  |  | 0.121 |
| Right Lung | 17 (40.48) | 30 (56.60) |  |
| Left Lung | 25 (59.53) | 23 (43.40) |  |
| Location_1 |  |  | 0.186 |
| Upper Lobe | 20 (47.62) | 32 (60.38) |  |
| Middle Lobe | 1 (2.38) | 2 (3.77) |  |
| Lower Lobe | 21 (50.00) | 19 (35.85) |  |
| Size (cm) | 4.55 ± 1.77 ^#^ | 3.40 ± 1.42 ^#^ | **0.001** |
| FERR (ng/mL) | 238.02 (187.80, 376.00) ^*^ | 173.79 (124.85, 288.00) ^*^ | **0.026** |
| SCCA (ng/mL) | 1.71 (0.93, 2.29) ^*^ | 0.77 (0.59, 0.93) ^*^ | **<0.001** |
| CA199 (U/mL) | 14.11 (6.36, 22.95) ^*^ | 16.36 (10.21, 19.89) ^*^ | 0.330 |
| AFP (ng/mL) | 2.37 (2.26, 3.38) ^*^ | 2.41 (2.34, 3.63) ^*^ | 0.941 |
| CEA (ng/mL) | 3.45 (2.68, 4.75) ^*^ | 4.39 (2.07, 10.84) ^*^ | **0.011** |
| CYFRA21-1 (ng/mL) | 4.29 (3.19, 6.62) ^*^ | 3.03 (2.48, 3.95) ^*^ | **0.014** |
| NSE (ng/mL) | 11.40 (10.18, 13.24) ^*^ | 11.10 (9.87, 12.79) ^*^ | 0.383 |

Note: Data in parentheses are percentages unless otherwise noted. BMI = Body Mass Index. FERR = ferritin. SCCA = squamous cell carcinoma antigen. CA = carbohydrate antigen. AFP = alpha-fetoprotein. CEA = carcinoembryonic antigen. CYFRA21-1= cytokeratin 19 fragment antigen. NSE = neuron specific enolase. ^#^ Values refer to mean ± standard deviation. ^*^Values refer to median (interquartile range). *P* values were the results of univariate analysis of each characteristic and the bold ones indicated statistical significance.

**Table S3** Comparison of selected radiomics features of prediction models between SCC and ADC patients in training and validation sets

| Characteristics | Training set (n=220) | | *p* | Validation set (n=95) | | *p* |
| --- | --- | --- | --- | --- | --- | --- |
|  | SCC (n=80) | ADC (n=140) |  | SCC (n=42) | ADC (n=53) |  |
| PET_Coarseness^GLNIDM^ | 0.03 (0.02, 0.04) | 0.05 (0.04, 0.07) | **<0.001** | 0.03 (0.02, 0.05) | 0.06 (0.03, 0.07) | **0.021** |
| PET_Strength^GLNIDM^ | 27.20 (15.79, 38.81) | 53.87 (40.65, 71.93) | **<0.001** | 30.67 (14.76, 51.70) | 58.04 (37.83, 76.40) | **0.007** |
| PET_ Normalized _Contrast^GLCM^ | 77.10 (64.35, 112.20) | 129.86 (99.85, 177.76) | **<0.001** | 92.60 (74.22, 132.22) | 147.07 (92.02, 219.74) | **0.002** |
| PET_ Normalized_Entropy^GLCM^ | 6.57 (6.17, 6.77) | 5.77 (5.20, 6.27) | **<0.001** | 6.38 (5.68, 6.66) | 5.64 (4.89, 6.35) | **0.003** |
| PET_SUV_min_ | 1.52 (1.18, 2.10) | 0.93 (0.70, 1.34) | **<0.001** | 1.26 (0.56, 1.73) | 0.93 (0.47, 1.33) | **0.043** |
| PET_SUV_mean_ | 7.11 (4.89, 8.62) | 3.58 (2.30, 5.68) | **<0.001** | 6.10 (3.88, 7.34) | 3.21 (2.32, 6.25) | **<0.001** |
| PET_Surface SUV_mean_ 1 | 5.70 (3.90, 7.12) | 3.13 (2.10, 4.80) | **<0.001** | 5.18 (3.57, 5.96) | 2.63 (2.10, 5.14) | **<0.001** |
| PET_Variance^TFC^ | 4.16 (3.52, 4.89) | 4.32 (3.50, 5.27) | **0.043** | 4.51 (3.33, 6.45) | 4.94 (3.70, 6.47) | **0.039** |
| PET_ Code Similarity^TFCCM^ | 0.14 (0.11, 0.18) | 0.09 (0.06, 0.14) | **<0.001** | 0.11 (0.06, 0.19) | 0.08 (0.06, 0.17) | **0.028** |
| PET_Entropy^NGLD^ | -1.33 (-1.83, -0.91) | -0.65 (-0.92, -0.40) | **<0.001** | -1.07 (-1.44, -0.56) | -0.56 (-0.98, -0.34) | **0.010** |
| CT_ Second angular moment^TFCCM^ | 0.15 (0.07, 0.38) | 0.07 (0.04, 0.16) | **<0.001** | 0.10 (0.02, 0.33) | 0.06 (0.03, 0.16) | **0.014** |
| CT_Correlation^GLCM^ | 0.81 (0.74, 0.86) | 0.76 (0.66, 0.81) | **<0.001** | 0.83 (0.70, 0.87) | 0.79 (0.71, 0.85) | **0.034** |
| CT_Asphericity | -46.95 (-49.74, -40.46) | -48.97 (-51.00, -44.13) | **0.001** | -56.14 (-60.46, -47.45) | -54.95 (-61.03, -49.34) | **0.028** |
| CT_Entropy_prod_surface_area | 56.64 (37.97, 87.76) | 25.29 (15.23, 40.62) | **<0.001** | 36.20 (16.74, 46.35) | 19.55 (9.76, 34.56) | **0.045** |
| CT_Entropy^NGLD^ | -3.43 (-3.95, -2.29) | -2.14 (-2.65, -1.61) | **<0.001** | -3.05 (-3.69, -2.40) | -2.30 (-3.31, -1.31) | **0.002** |

Note: GLNIDM = Gray Level Neighborhood Intensity-difference Matrix. GLCM = Gray Level Co-occurrence Matrix. TFCCM = Texture Feature Coding co-occurrence matrix. NGLD = Neighboring Gray Level Dependence. TFC = Texture Feature Coding. Values refer to median (interquartile range). *P* values were the results of univariate analysis of each characteristic and the bold ones indicated statistical significance.
